# The Effect of Conductive Heat Transfer on the Morphology Formation in Polymer Solutions Undergoing Thermally Induced Phase Separation

**DOI:** 10.3390/polym14204345

**Published:** 2022-10-15

**Authors:** Samira Ranjbarrad, Philip K. Chan

**Affiliations:** Department of Chemical Engineering, Toronto Metropolitan University, 350 Victoria Street, Toronto, ON M5B 2K3, Canada

**Keywords:** thermally induced phase separation, nonisothermal, spinodal decomposition, temperature gradient, enthalpy of demixing

## Abstract

Owing to the fact that heat transfer during the thermally induced phase separation process is limited, a quench rate is inevitably entailed, which leads to the existence of temporal and spatial variations in temperature. Hence, it is of great importance to take into account the nonisothermality during the phase separation process, especially in high viscosity polymer solutions. In this study, the influence of conductive heat transfer on the morphology formation during the thermally induced phase separation process was investigated theoretically in terms of quench depth, boundary conditions, and enthalpy of demixing to elucidate the interaction between temperature and concentration through incorporating the nonlinear Cahn-Hilliard equation and the Fourier heat transfer equation in two dimensions. The Flory-Huggins free energy theory for the thermodynamics of phase separation, slow mode theory, and Rouse law for polymer diffusion without entanglements were taken into account in the model development. The simulation results indicated a strong interaction between heat transfer and phase separation, which impacted the morphology formation significantly. Results confirmed that quench depth had an indispensable impact on phase separation in terms of higher characteristic frequency by increasing the driving force for heat transfer. Applying quench from various boundaries led to a difference in the quench rate due to the high viscosity of the polymer solution. This led to a gradation in pore size and anisotropic morphology formation. The degree and direction of anisotropy depended on quench depth and rate, quench time, heat conduction rate inside the solution, solution viscosity, temperature evolution, and the enthalpy of demixing. It was also verified that the influence of enthalpy of demixing on phase separation could not be neglected as it increased the solution temperature and led to phase separation being accomplished at a higher temperature than the initial quench temperature.

## 1. Introduction

The thermally induced phase separation process (TIPS) is one of the principal methods of producing numerous functional polymeric materials that are widely employed in industrial applications, such as membranes, microcellular foams, thermally reversible porous gels, and polymer-dispersed liquid crystals [1,2,3,4,5,6,7,8]. During the TIPS process, the quality of the solvent decreases through an instantaneous quench in systems with an upper critical solution temperature (UCST) or an immediate jump in temperature in systems with a lower critical solution temperature (LCST) [4,9,10,11,12,13,14]. In systems with an UCST, a low-molecular-weight high boiling point solvent is initially mixed with a polymer at an elevated temperature to form a homogenous mixture [15,16,17,18]. The temperature drops suddenly to induce phase separation by bringing the system into the two-phase region of the phase diagram where the system phase separates into polymer-rich and polymer lean phases [1,7,19,20,21]. In the final step, the medium is frozen via crystallization, gelation, or vitrification, and the solvent may be extracted to obtain the desired morphology [2,22,23,24,25]. Spinodal decomposition is categorized into three principal stages of early, intermediate, and late stages based on the time evolution of the sinusoidal waves that specify the one-dimensional spatial concentration fluctuations [1,2,8,26,27]. In the early stage, the amplitude of the fluctuations increases with time while the wavelength of the spatial concentration fluctuations stays constant. The wavelength and amplitude both grow with time during the intermediate stage. In the late stage of the spinodal decomposition, the amplitude of the fluctuations reaches its equilibrium value while the wavelength increases with time as a result of coarsening [1,13,28,29,30].

The widespread applicability of the materials produced through the TIPS process has prompted researchers to investigate the process dynamics and the parameters influencing the phase separation [1,7,8,31,32,33,34]. The characteristics of the porous polymeric materials, such as final morphology, pore size, and shape, can be controlled in the TIPS process, by regulating the operating parameters and a deep knowledge of the dynamics and thermodynamics of the phase separation process [7,10,18,35]. Producing anisotropic morphologies is one of the most prominent features of the TIPS process [36,37,38,39,40,41]. An electrical field, a temperature gradient, a shear flow, a chemical reaction, or a concentration gradient can all be employed to create anisotropy [2,17,18,42,43,44]. A deep understanding of the impact of these techniques to induce anisotropy leads to in-depth knowledge of the fabrication of functional polymeric materials with desired morphologies, and mechanical, optical, and thermal properties [2,6,45,46,47,48].

In the majority of theoretical investigations of the TIPS process, the process is presumed to be isothermal, which implies that the system is quenched to a constant temperature and is maintained at that temperature with no variations in space and time [30,49,50,51,52,53]. Several attempts have been made to study the TIPS process considering a temperature gradient [1,2,22,31,50,54,55,56]. Caneba and Soong [16,31] were the first researchers to provide a noteworthy description of the spinodal decomposition through experimental and modelling studies of the coupled spinodal decomposition and heat transfer in one dimension for a critical quench of polymethyl methacrylate (PMMA)/sulfolane solution using the thermal inversion process. A temperature gradient with spatial and temporal variations was created in the direction of the applied quench. The development of smaller droplets on the quench side and larger pores on the insulated side was confirmed. Ullmann et al. [22] investigated the influence of heat transfer during phase separation through spinodal decomposition in the deep quenched critical mixtures. They confirmed heat transfer enhancement in systems with an UCST through coupling mass, momentum, and energy conservation equations. They also discovered that raising the specific heat improved phase separation by minimizing the effect of the heat of demixing generated during phase separation. The enthalpy of demixing, or the heat released during phase separation, slowed down the process by raising the quench temperature, resulting in phase separation at higher temperatures. In a series of papers, Molin and Mauri [33,57] used mass, momentum, and energy conservation equations to model the phase separation of a critical solution, considering heat transfer rates during phase separation. Poesio et al. [58] explored the heat transfer enhancement during convection-driven spinodal decomposition experimentally, comparing the heat transfer rates in the case of convection versus no convection, and found that the convective motion greatly reduced the cooling time. Matsuyama et al. [40] investigated the influence of quench temperature, quenching medium, and cooling rate on the generation of asymmetric and anisotropic membranes during the TIPS process. They induced phase separation by evaporating the solvent from one side of the membrane and immersing the membrane in ice water from the other side, and they discovered that the top part of the polymer solution, which was partially evaporated, had smaller pores, whereas the bottom of the membrane with lower polymer concentration had larger pores. Atkinson and Lloyd [25] added temperature-dependent variables to their model. This allowed them to quantify the how temperature affected weight loss, cell size, and concentration fluctuations.

Using a time-dependent temperature profile and a growth law expression for the late stage of phase separation, Barton and McHugh [27] provided a model based on the Cahn-Hilliard theory for droplet formation near the glass transition temperature of the polymethyl methacrylate (PMMA)/cyclohexanol polymer solution. They indicated that a temperature gradient caused an anisotropic membrane formation, with the degree of anisotropy being time-dependent. In a series of papers, Lee et al. [1,2] investigated the morphology formation during phase separation numerically using the TIPS process with a spatial temperature gradient. They observed anisotropic morphology as a result of an external temperature gradient. The direction of anisotropy was demonstrated to be time-dependent, implying that if a short period of time was given for phase separation, small droplets would develop in the lower temperature regions. The Ludwig-Soret effect, in which a concentration gradient due to phase separation parallel or anti-parallel to the temperature gradient forms in a thermally induced phase separation process with a spatial temperature gradient, was theoretically investigated by Kukadiya et al. [13]. Results demonstrated that the Ludwig-Soret effect had a minor impact on the mechanism of phase separation in the presence of a non-uniform temperature gradient. Tabatabaieyazdi et al. [20,59,60] investigated short-range, long-range, and multiple surface directed phase separation processes under a linear temperature gradient in polymer blends. To inspect the anisotropic membrane formation of a polymer solution theoretically, Hong and Chan [4] inspected the simultaneous concentration and temperature gradients on the morphology formation during the TIPS process. They concluded that the direction of the temperature and concentration gradients was the determining factor in the anisotropic morphology formation during the thermally induced phase separation process. Huston et al. [54] investigated the phase separation through spinodal decomposition with continuous cooling using the temperature-dependent coefficients in the Cahn-Hilliard equation and extended their model to include two scenarios of fast and slow cooling. Cervellere et al. [61] proposed a phase-field model for the TIPS process of polyvinylidene fluoride/diphenyl carbonate solution and simulated their model in 3D for isotropic and anisotropic quenches. A thicker layer at the cooling surface was discovered, the thickness of which was determined by the quench temperature, polymer volume fraction, and thermal conduction rate through the solution.

During the actual TIPS process, temperature varies in space and time, and a quench rate is always involved as a result of the limited heat transfer especially in high-viscosity polymer solutions [24,61,62]. This study is intended to elucidate the influence of nonisothermality on the phase separation in terms of quench depth, boundary conditions and enthalpy of demixing. Coupling heat and mass transfer will lead us to understand the dynamics and thermodynamics of the phase separation process more precisely. To the best of our knowledge, there is no reported study on the influence of the two-dimensional temporal and spatial variations of temperature during the thermally induced phase separation process for anisotropic morphology formation considering the influence of the enthalpy of demixing. In this study, numerical analysis of the combined effect of the transient heat and mass transfer and its influence on phase separation were accomplished through taking into account the temperature-dependent parameters in the Cahn-Hilliard equation [49]. The model utilized incorporated the nonlinear Cahn-Hilliard theory using the Flory-Huggins free energy density for the thermodynamics of the phase separation process. In addition, the Rouse model and slow-mode theory were used to characterize polymer diffusion in an unentangled polymer solution of polystyrene-cyclohexanol [15,42,63]. Phase separation of the binary mixture was simulated in a domain with walls being quenched below the critical temperature from all four sides and from one side. It is worth noting that diffusion and heat transfer occur at different time scales. Diffusion is substantially slower than the thermal transition. However, the dynamics of temperature change in the system affects the spatial and temporal pattern development [16,55].

## 2. Model Development

The model development for the nonisothermal thermally induced phase separation process is described in this section. The two-dimensional Fourier heat transfer equation and the Cahn-Hilliard equation were coupled in a square geometry. The nonlinear Cahn-Hilliard equation was utilized to determine the dynamics of phase separation in the unstable region of the phase diagram where phase separation was accomplished through spinodal decomposition. The Cahn-Hilliard model considers an isothermal state of phase separation [19,30,49] which implies that the whole system is supposed to be in contact with a heat reservoir, and as a result, the thermal fluctuations are neglected with no energy conservation limitations [51]. In the Cahn-Hilliard theory, the total free energy of a heterogeneous binary mixture that undergoes phase separation through spinodal decomposition is defined using Equation (1) as [30]:

(1) $$F=\int \left[f\left(c\right)+\kappa {\left(\nabla c\right)}^{2}\right]dV$$

where $f\left(c\right)$ is the free energy of the homogenous system, κ is the positive interfacial gradient energy coefficient, and c is the concentration of the solvent in terms of volume fraction. The second term in the integral accounts for the concentration gradient due to phase separation. The model that was employed to describe the thermodynamics of phase separation through spinodal decomposition was the Flory-Huggins theory, which is capable of describing the thermodynamics of phase separation in polymer solutions very successfully [16]. Although Flory-Hugging’s theory has some limitations, it is a very effective theory in the phase equilibrium investigations [1,6,8,15,20,44]. The free energy of mixing of the homogenous system in the Flory-Huggins theory is defined as [64]:

(2) $$f\left(c\right)=\frac{k_{B}T}{\nu }\left[\frac{c}{N_{1}}\mathrm{lnc}+\frac{1-c}{N_{2}}\ln\left(1-c\right)+\chi c\left(1-c\right)\right]$$

where *T* is the temperature, *v* is the volume of the cell or segment of the polymer, χ is Flory’s interaction parameter, *k_B_* is the Boltzmann’s constant, *N*_1_ and *N*_2_ are the degrees of polymerization of the solvent and the polymer, respectively. In polymer solutions, the degree of polymerization of the solvent *N*_1_ is 1 and the degree of polymerization of the polymer *N*_2_, was set to 100 in this study. The polar interactions and hydrogen bonding were considered in calculating Flory’s interaction parameter. The correlation that was utilized to determine the interaction parameter is the one used by Nistor et al. [47] in the simple thermodynamics of a binary mixture defined as [6,47]:

(3) $$\chi =\frac{V_{\mathrm{CHOL}}}{RT}{\left(\delta_{\mathrm{CHOL}}-\delta_{\mathrm{ps}}\right)}^{2}+0.34$$

where *V*_CHOL_ is the molar volume of cyclohexanol and δ is the Hildebrand solubility parameter. It is beneficial to define the interaction of the polymer and the solvent through the solubility parameter. Hansen’s contribution theory, which is a useful explanation of polarity, designates the solubility parameter to depend on polar interactions ($\delta_{p}$), hydrogen bonding ($\delta_{h}$) and the dispersive term ($\delta_{d}$) in terms of [65]:

(4) $$\delta_{\mathrm{PS}}{}^{2}=\delta_{p}{}^{2}+\delta_{h}{}^{2}+\delta_{d}{}^{2}$$

The solubility parameters can be obtained from polymer textbooks [66,67,68]. The temperature dependence of the solubility parameter was neglected due to the fact that the change of the solubility parameter as a result of the change in temperature in the range of the temperature utilized in this study was not significant. The nonlinear Cahn-Hilliard equation was derived through the continuity equation in the form of [30]:

(5) $$\frac{\partial c}{\partial t}=-\nabla •j$$

where **j** is the interdiffusional flux determined as a function of chemical potential gradient:

(6) $$j=-M\;\nabla \;\left(\mu_{2}-\mu_{1}\right)=-M\frac{\delta F}{\delta c}$$

where *M* is the mobility that is related to the concentrations and molecular weights of the polymer and the solvent and $\mu_{1}$ and $\mu_{2}$ are the chemical potentials of component 1 and 2.

The nonlinear Cahn-Hilliard equation was derived by combining Equations (1), (5), and (6) [69,70]:

(7) $$j\frac{\partial c}{\partial t}=\nabla \cdot \left[M\nabla \left[\frac{\partial f}{\partial c}-2\kappa \nabla^{2}c\right]\right]$$

The mobility was defined through the self-mobilities of the components based on the slow-mode theory, which requires more time for phase separation and is a more appropriate choice for the slow diffusion process in high-viscosity polymer solutions defined as: [15,63]

(8) $$\frac{1}{M}=\frac{1}{M_{1}}+\frac{1}{M_{2}}$$

*M*_1_ and *M*_2_ are the self-mobility of the solvent and polymer, respectively.

The self-mobility and self-diffusivity of each component are related to each other based on the following equation:

(9) $$D_{i}=M_{i}(\frac{\partial^{2}f}{\partial c_{i}^{2}})$$

The self-diffusion coefficient is related to the degree of polymerization through the Rouse model for unentangled polymer chains with *N_i_* < 200 and is expressed as [71,72,73]:

(10) $$D_{i}=\frac{k_{B}T}{\xi_{i}N_{i}},\;\mathrm{for}\;i=1,\;2$$

$\xi_{i}$ is the frictional coefficient per cell or segment of the polymer or solvent molecule. *c*_1_ is the volume fraction of the solvent, and *c*_2 =_ 1 − *c*_1_ is the volume fraction of the polymer. If frictional coefficients of the solvent and the polymer are assumed to be equal and independent of concentration and temperature ($\xi_{1}=\xi_{2}=\xi$), and no interaction exists between polymer and solvent, the mobility is defined through the following equation as [12,15,72]:

(11) $$M=\frac{\nu c\left(1-c\right)}{\xi }$$

In order to determine the gradient energy coefficient, Debye’s thermodynamic theory for nonhomogeneous solutions was utilized [74,75]:

(12) $$\kappa =\frac{RT\chi l^{2}}{6}$$

where l is the molecular interaction length between the solvent and the polymer and χ is the Flory’s interaction parameter. Lastly, the scaling relations below were utilized to nondimensionalize the mathematical model developed in this paper:

(13) $$c^{*}=c$$

(14) $$T^{*}=\frac{T}{\theta }$$

(15) $$x^{*}=\frac{x}{L}$$

(16) $$y^{*}=\frac{y}{L}$$

(17) $$t^{*}=\frac{k_{B}\theta \;R_{g}^{2}}{\zeta L^{4}}t$$

(18) $$c_{p}{}^{*}=\frac{\rho c_{p}\nu }{k_{B}}$$

(19) $$\lambda =\frac{\alpha \zeta L^{2}}{k_{B}\theta \;R_{g}^{2}}=\frac{\alpha \Lambda }{D}=Le*\Lambda$$

(20) $$l^{*}=\frac{l}{L}$$

(21) $$\Lambda =\frac{L^{2}}{\;NR_{g}^{2}}$$

where *L* is the length of the sample in the dimension of the square *L × L* and was set to 20 µm in this study. t* is the dimensionless time and c_p_* is the dimensionless specific heat. The two-dimensional Cahn-Hilliard equation and the Fourier heat transfer equation were derived using the scaling relations. Two dimensionless numbers were introduced in this study. The first one was Λ which was the square of the medium length over the product of the square of the radius of gyration and the degree of polymerization. This dimensionless number was defined as the dimensionless characteristic length. This parameter denotes the length of the sample with respect to the size of the polymer chain. The second dimensionless parameter λ was a combination of Λ, heat diffusivity α, and the diffusion coefficient D. This dimensionless parameter can be explained as the combination of Lewis number *Le* which describes the rate of temperature spread through the material to the diffusion coefficient, and the Λ parameter. Hence, it provides information on the rate of heat diffusion with respect to polymer diffusion. The rate of heat conduction inside the solution influences the morphology formation as well. If heat diffusion is faster than the diffusion of mass, as is the case for high viscosity polymer solutions, heat will diffuse faster and reach the homogenous temperature in the entire sample before phase separation is completed.

**Figure 1:**
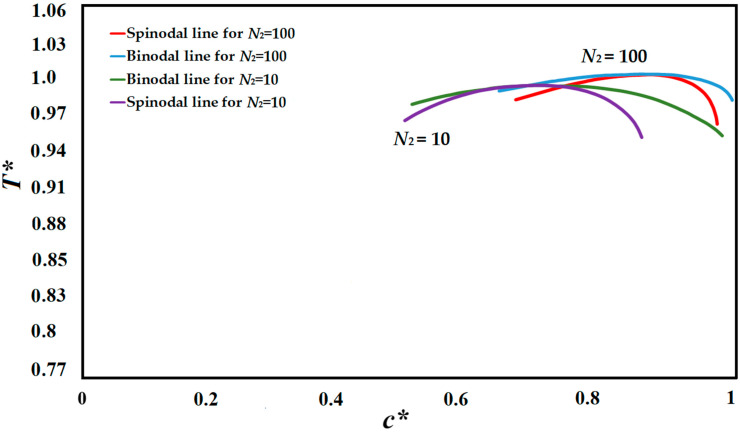
Schematic phase diagram of the polystyrene-cyclohexanol polymer solution with two degrees of polymerization of *N*_2_ = 10 and *N*_2_ = 100.

The compatibility of the polymer solution is defined via a phase diagram, which designates the range and phase boundaries appropriate for phase separation as a function of concentration and temperature [9,76]. Cooling rate and quench time, as well as composition and temperature, are the major factors in defining the phase separating path [9,76]. The polymer solution utilized in this study was the high viscosity polystyrene-cyclohexanol polymer solution. The phase diagrams of the polystyrene-cyclohexanol polymer solution for the degrees of polymerization of *N*_2_ = 10, and *N*_2_ = 100 are presented in Figure 1 which are plotted using the Flory-Huggins theory and are in accordance with the experimentally and theoretically obtained phase diagram [6,28,40,47,76,77,78]. *N*_2_ is the degree of polymerization of the polymer and *N*_1_ is the degree of polymerization of the solvent, which is *N*_1_ = 1. The simulation results provided in this study are based on *N*_2_ = 100.

The governing Cahn-Hilliard equation in terms of the dimensionless quantities is provided as follows:

(22) $$\begin{array}{ll} \frac{\partial c^{*}}{\partial t^{*}}=\Lambda T^{*}(\frac{1}{N_{2}} & -1-2\chi \left(1-2c^{*}\right))(\frac{\partial c^{*}}{\partial x^{*}}\frac{\partial c^{*}}{\partial x^{*}}+\frac{\partial c^{*}}{\partial y^{*}}\frac{\partial c^{*}}{\partial y^{*}})\\ & +\Lambda T^{*}\left(\left(1-c^{*}+\frac{c^{*}}{N_{2}}-2\chi \left(1-2c^{*}\right)\right)\left(\frac{\partial^{2}c^{*}}{\partial x^{*}{}^{2}}+\frac{\partial^{2}c^{*}}{\partial y^{*}{}^{2}}\right)\right)\\ & -\frac{T^{*}\chi }{3}\left(1-2c^{*}\right)\left\{\frac{\partial c^{*}}{\partial x^{*}}\left(\frac{\partial^{3}c^{*}}{\partial x^{*}{}^{3}}+\frac{\partial^{3}c^{*}}{\partial x^{*}\partial y^{*}{}^{2}}\right)+\frac{\partial c^{*}}{\partial y^{*}}\left(\frac{\partial^{3}c^{*}}{\partial y^{*}{}^{3}}+\frac{\partial^{3}c^{*}}{\partial y^{*}\partial x^{*}{}^{2}}\right)\right\}\\ & -\frac{T^{*}\chi }{3}c^{*}\left(1-c^{*}\right)\left(\frac{\partial^{4}c^{*}}{\partial x^{*}{}^{4}}+\frac{2\partial^{4}c^{*}}{\partial x^{*}{}^{2}\partial y^{*}{}^{2}}+\frac{\partial^{4}c^{*}}{\partial y^{*}{}^{4}}\right) \end{array}$$

The most suitable initial condition for solving the Cahn-Hilliard equation is to consider the infinitesimal thermal concentration fluctuations that are present in the homogenous polymer solution. The dimensionless expression for the initial concentration is provided as [15]:

(23) $$c^{*}\left(t^{*}=0\right)=c^{*}{}_{0}+\delta c^{*}\left(t^{*}=0\right)$$

$c^{*}$ is the dimensionless average concentration of the solvent and $\delta c^{*}$ presents the initial concentration fluctuations of the system at thermal equilibrium which was considered to be $\pm 10^{-6}$ in this paper.

The nonperiodic boundary conditions were considered to provide a more realistic simulation of the phase separation process. Due to the fact that no mass was transferred through the surfaces, the zero-mass flux boundary condition was a suitable choice. The zero-mass flux boundary conditions used in this study are as follows [15,63]:

(24) $$\frac{\partial^{3}c^{*}}{\partial x^{*}{}^{3}}+\frac{\partial^{3}c^{*}}{\partial x^{*}{}^{2}\partial y^{*}{}^{2}}=0,\;\mathrm{at}\;t^{*}>0\;\mathrm{and}\;x^{*}=0\;\mathrm{and}\;x^{*}=1$$

(25) $$\frac{\partial^{3}c^{*}}{\partial y^{*}{}^{3}}+\frac{\partial^{3}c^{*}}{\partial y^{*}{}^{2}\partial x^{*}{}^{2}}=0,\;\mathrm{at}\;t^{*}>0\;\mathrm{and}\;x^{*}=0\;\mathrm{and}\;x^{*}=1$$

Furthermore, natural boundary conditions that were gained through the change in free energy were selected as the other set of boundary conditions. The natural boundary conditions used in this paper are as follows [15,63]:

(26) $$\left[\nabla^{*}c^{*}\right]•\;n=0$$

(27) $$\frac{\partial c^{*}}{\partial x^{*}}=0,\;\mathrm{at}\;t^{*}>0\;\mathrm{and}\;x^{*}=0\;\mathrm{and}\;x^{*}=1$$

(28) $$\frac{\partial c^{*}}{\partial y^{*}}=0,\;\mathrm{at}\;t^{*}>0\;\mathrm{and}\;x^{*}=0\;\mathrm{and}\;x^{*}=1$$

The temporal and spatial temperature gradients were considered through the Fourier heat transfer equation in the *x*- and *y* directions as follows [79]:

(29) $$\frac{\partial T^{*}}{\partial t^{*}}=\lambda \left(\frac{\partial^{2}T^{*}}{\partial x^{*}{}^{2}}+\frac{\partial^{2}T^{*}}{\partial y^{*}{}^{2}}\right)+\dot{q}$$

where $\dot{q}$ is the energy dissipation term which was considered as the enthalpy of demixing [33,57,79]. The difference in the mixing enthalpy between the initial system and the demixed system was defined through the Flory-Huggins theory and the lever rule for a system with initial polymer composition *c*_0_ and the phase separated compositions of *c*_1_ and *c*_2_ as [28,80,81,82,83]:

(30) $$\Delta H_{\mathrm{demix}}=\frac{k_{B}T\chi }{\nu (c_{2}-c_{1})}\left[\begin{array}{c} (c_{2}-c_{0})\left(1-c_{1}\right)+(c_{0}-c_{1})c_{2}\left(1-c_{2}\right)\\ -(c_{2}-c_{1})c_{0}\left(1-c_{0}\right) \end{array}\right]$$

To solve the Fourier heat transfer equation, the medium was initially considered at an elevated initial solution temperature in a homogenous state as the initial condition. Two sets of boundary conditions were applied in order to investigate the morphology formation as a result of various quench conditions, i.e., applying quench from all four sides of the medium, and applying quench from one side and keeping the other sides insulated. The boundary conditions are summarized in Table 1. The initial solution temperature was defined as T*, the quench temperature was termed as T*_q_ and the initial solution concentration was demonstrated as c*. The finite element method was applied to obtain the set of time-dependent ordinary differential equations (ODEs) that were solved by the Newton-Raphson method. The Galerkin finite element method was utilized to solve the governing equations using the Hermitian basis function to discretize space [15,63]. A mesh of 80 × 80 was considered to discretize the square domain. As there were 8 values per node, the size of the Jacobian matrix was 52,448 × 52,448. The Forward-Backward Euler method was employed for the time integration. The convergence criterion was set to be the difference between the two consecutive solutions being less than 10^−6^. The Fortran programming language was utilized to simulate the process, and each simulation was performed for almost two weeks using the high-performance computers in the graduate computer lab of the Chemical Engineering Department, Toronto Metropolitan University and Digital Research Alliance of Canada.

## 3. Results and Discussion

In this section, the simulation results are provided and compared for various initial average concentration and quench depths listed in Table 1. It should be noted that, the number of simulations that have been conducted is substantial. However, the results that elucidate the dynamics and thermodynamics of the process more precisely to achieve the objectives of the current paper are provided in this paper.

In the following simulation results, the initial volume fraction was kept constant with the quench temperature being categorized into three different quench depths, termed as cases 1–4 in Table 1, and the influence of the quench depth on the morphology formation during the TIPS process was investigated. In all cases, the direction of the temperature gradient was normal to the walls of the medium, and the temporal and spatial variations of the temperature gradient inside the sample and its influence on phase separation and morphology formation were inspected.

**Figure 2:**
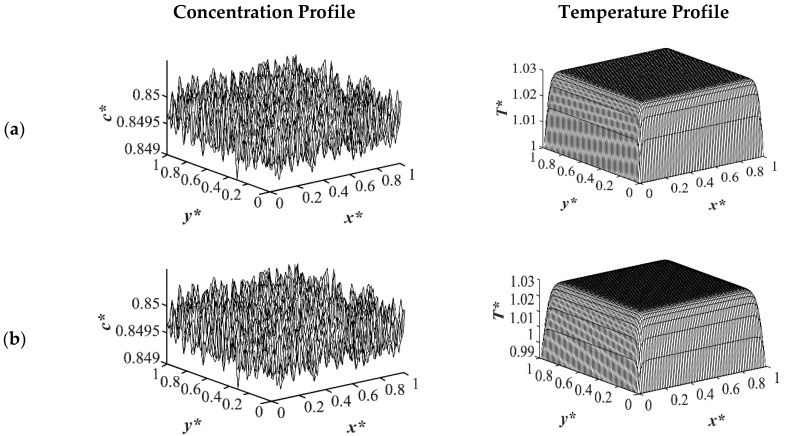

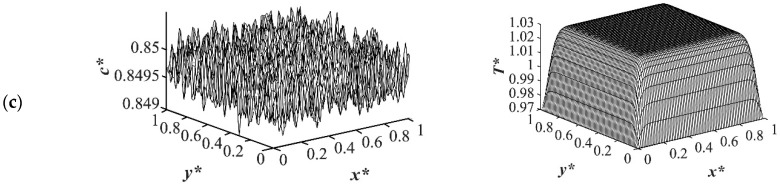
Spatial concentration (**left** column) and temperature (**right** column) profiles for the off-critical quench to c* = 0.85 at t* = 1.03 × 10^−6^ for three different quench depths of (**a**) T*_q1_ = 1.0, (**b**) T*_q2_ = 0.99 (**c**) T*_q3_ = 0.97.

**Figure 3:**
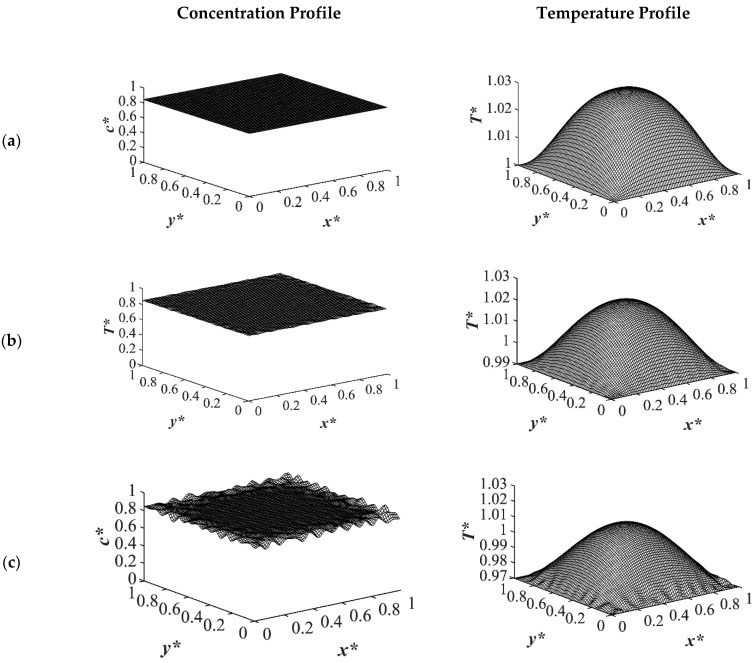
Spatial concentration (**left** column) and temperature (**right** column) profiles for the off-critical quench to c* = 0.85 at t* = 3.03 × 10^−5^ for three different quench depths of (**a**) T*_q1_ = 1.0, (**b**) T*_q2_ = 0.99 (**c**) T*_q3_ = 0.97.

**Figure 4:**
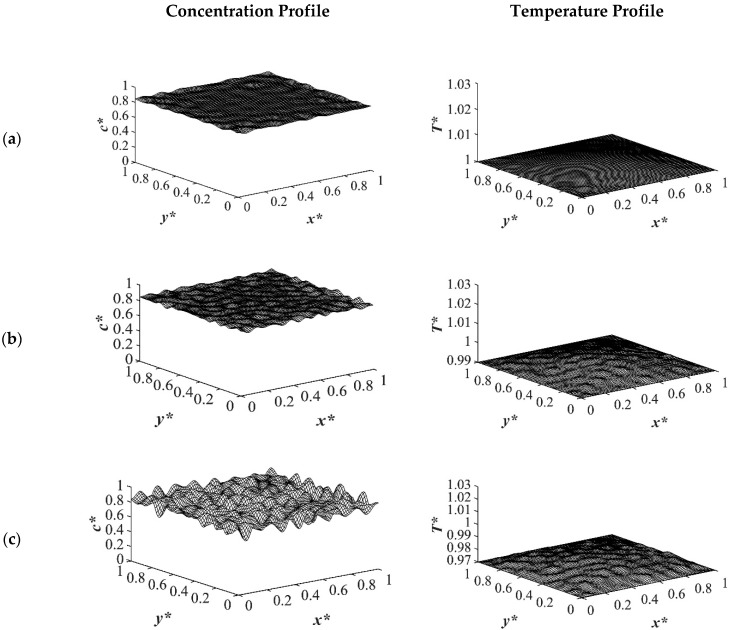
Spatial concentration (**left** column) and temperature (**right** column) profiles for the off-critical quench to c* = 0.85 at t* = 2.03 × 10^−4^ for three different quench depths of (**a**) T*_q1_ = 1.0, (**b**) T*_q2_ = 0.99 (**c**) T*_q3_ = 0.97.

**Figure 5:**
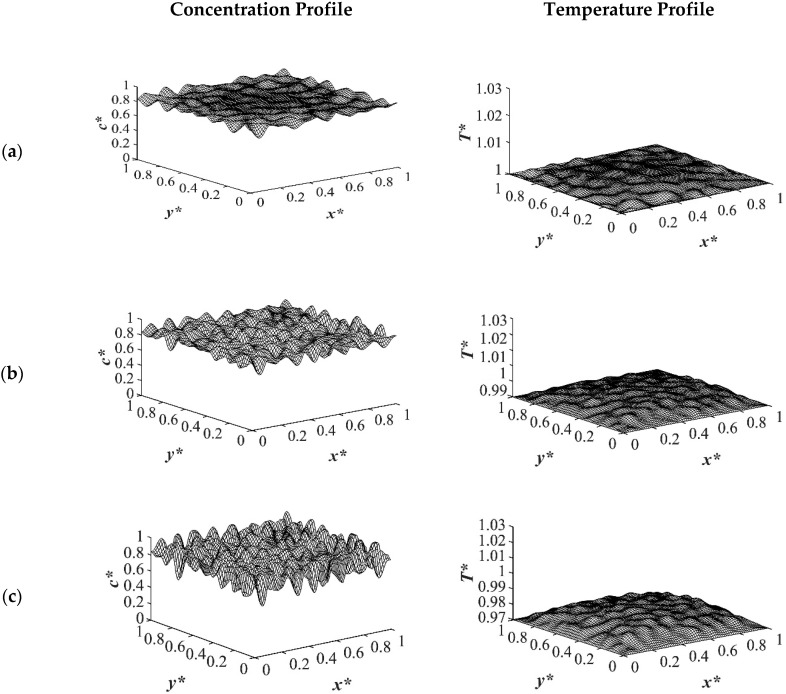
Spatial concentration (**left** column) and temperature (**right** column) profiles for the off-critical quench to c* = 0.85 at t* = 8.03 × 10^−4^ for three different quench depths of (**a**) T*_q1_ = 1.0, (**b**) T*_q2_ = 0.99 (**c**) T*_q3_ = 0.97.

The results of case 1 of applying three different quench temperatures of T*_q3_ = 0.97, T*_q2_ = 0.99 and T*_q1_ = 1.0 from all four sides of the sample are provided in Figure 2, Figure 3, Figure 4 and Figure 5 for an off-critical quench with a dimensional initial concentration of c* = 0.85 and the influence of the quench depth on morphology formation was evaluated at different times during phase separation. The initial dimensionless solution temperature was set to T* = 1.03. The dimensionless concentration profiles are presented on the left side, and the corresponding temperature profiles are presented in the right column. Most of the experimental studies and industrial processes conducted for polymeric materials’ formation using the TIPS process, apply quench to one or two sides of the medium. The results provided in this section go one step further and provide a qualitative analysis of the morphology formation when all four sides of the sample are quenched to the same temperature. The dimensionless concentration c* as a function of x* and y* at time t^*^ = 1.03 × 10^−6^ and the corresponding dimensionless temperature T* profile are presented in Figure 2. Phase separation did not initiate at this time yet and the infinitesimal thermal concentration fluctuations that were specified in the initial condition of the Cahn-Hilliard equation can be observed in the concentration profile for all quench depths. However, the temperature profiles are different at different quench depths, which indicates that increasing quench depth leads to higher driving force for heat transfer. The transient temperature evolves faster than the phase separation upon applying quench in the high-viscosity polymer solution.

As the time proceeded to t* = 3.03 × 10^−5^, as shown in Figure 3, the morphology started to evolve for the deep quench to T*_q3_ = 0.97 from all four sides of the sample and advanced inside the medium gradually. However, no phase separation was accomplished for the shallow quench to T*_q1_ = 1.0 yet. Due to the low interfacial tension between the two phases at this quench depth, the difference in the equilibrium concentrations between the two phases was not significant, which led to a lower phase separation rate. The dimensionless concentration profiles for the quench depths of T*_q2_ = 0.99, and T*_q3_ = 0.97 showed phase separation initiation from all four sides, which is more evident in the deep quench to T*_q3_ ^=^ 0.97. Anisotropic morphology would result due to the difference in the quench rate in the boundaries and inside the sample. Although there was a variation in the droplet size between shallow and deep quenches, due to the polar interactions and the hydrogen bonding effects of the solvent, the morphology difference between the shallow and deep quenches was not substantial, as verified by Nistor et al. [6]. The major difference between the shallow and deep quenches was the time it took for the entire system to phase separate.

The difference between the shallow and deep quenches starts to emerge at t* = 2.03 × 10^−4^ as shown in Figure 4. As is evident, the temperature reached almost its homogenous state while the phase separation was still progressing. However, the inhomogeneity in the dimensionless temperature profiles was due to the enthalpy of demixing effect, which will be discussed further. The number of the droplets increased as the quench depth increased, or the quench temperature decreased. On the other hand, the size of the droplets decreased as the quench depth increased even though the change was not considerable. The dimensionless temperature profile presented in Figure 4a shows that the temperature has almost become homogenous in the entire sample with a very low amount of heat released. Whereas the temperature profiles presented in Figure 4b,c show more heat being released during the phase separation process. The four sides of the medium reached the intermediate stage of phase separation at t* = 2.03 × 10^−4^ while the interior parts were still in the initial stage due to the high viscosity of the solution that prevented convection driven heat transfer. Heat did not propagate fast enough to induce phase separation homogenously, and interior parts of the sample were still at higher temperatures than the quench sides, which led to slower phase separation rates. This led to gradation in the pore size due to the difference in the quench rate between various parts of the sample, and hence anisotropic morphology resulted. The heat that was released during phase separation performed as a shallow quench effect and increased the quench temperature, which led to phase separation being accomplished at a higher temperature than the initial quench temperature. Although this increase in temperature is not significant in polymer solutions and blends, it is verified in this study that it cannot be neglected. The comparison between the temperature profiles from the shallowest to the deepest quench revealed that increasing the quench depth led to higher rates of heat transfer and, consequently, higher rates of phase separation by increasing the driving force for heat transfer and phase separation due to the difference in the compositions between the two phases.

Figure 5 displays the dimensionless concentration and temperature profiles at t* = 8.03 × 10^−4^. In the case of deep quench to T*_q3_ = 0.97, more phase separation was accomplished, and phase separation reached its final stage on the four sides where the droplets started to coarsen by merging while the interior parts were still in the intermediate stage of phase separation. A comparison between the deep and shallow quenches reveals that there is a significant difference between the shallow quench to T*_q1_ = 1.0 and the deep quench to T*_q3_ = 0.97 which led to more phase separation being performed in the deep quench with smaller droplet formation. As the quench depth increased, the driving force for heat transfer increased.

Phase separation proceeded in the entire sample with droplets of smaller sizes formed on its four sides. This was due to the difference in the quench rate between the boundaries and inside the sample. The solution viscosity was higher at c* = 0.85 in comparison with other concentrations investigated, which decreased the rate of heat transfer and hence impacted the morphology formation noticeably. In addition, applying deeper quench led to an increase in the solution viscosity, which is temperature-dependent and caused an increase in the phase separation time. This was verified by Nistor et al. [6] and Song and Torkelson [8] for the polystyrene-cyclohexanol [6,47]. This was irrespective of the quench depth and occurred in all cases because the gradient in the quench rate existed between the sample boundaries and inside the sample in all three cases.

At the deep quench to T*_q3_ = 0.97, the direction of anisotropy would change and the smaller droplets that were formed in the boundaries would merge due to coarsening, which would increase the size of the droplets in comparison with the interior parts. This was previously verified by Lee et al. [1,2] as a result of applying a linear temperature gradient in the sample.

**Figure 6:**
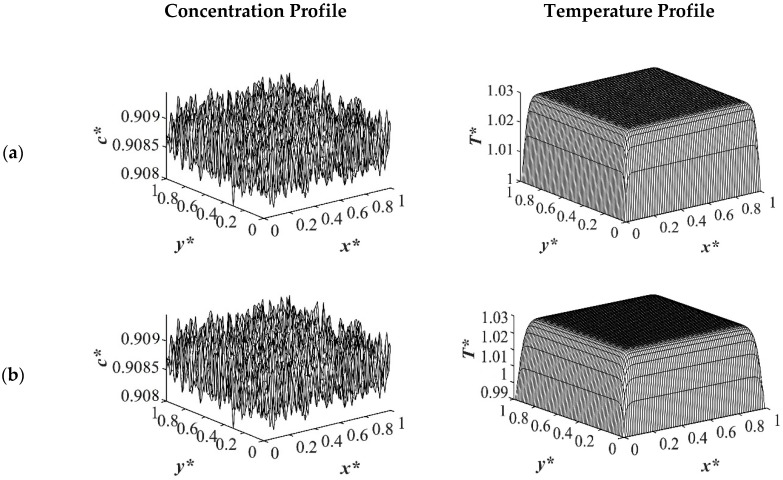

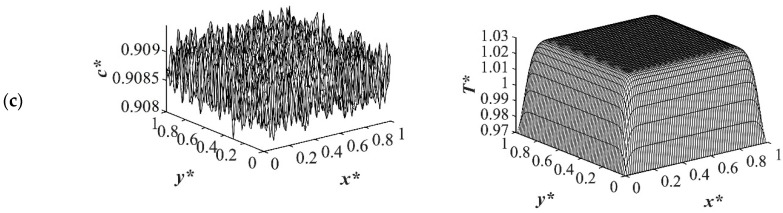
Spatial concentration (**left** column) and temperature (**right** column) profiles for the off-critical quench to c* = 0.909 at t* = 1.03 × 10^−6^ for three different quench depths of (**a**) T*_q1_ = 1.0, (**b**) T*_q2_ = 0.99 (**c**) T*_q3_ = 0.97.

**Figure 7:**
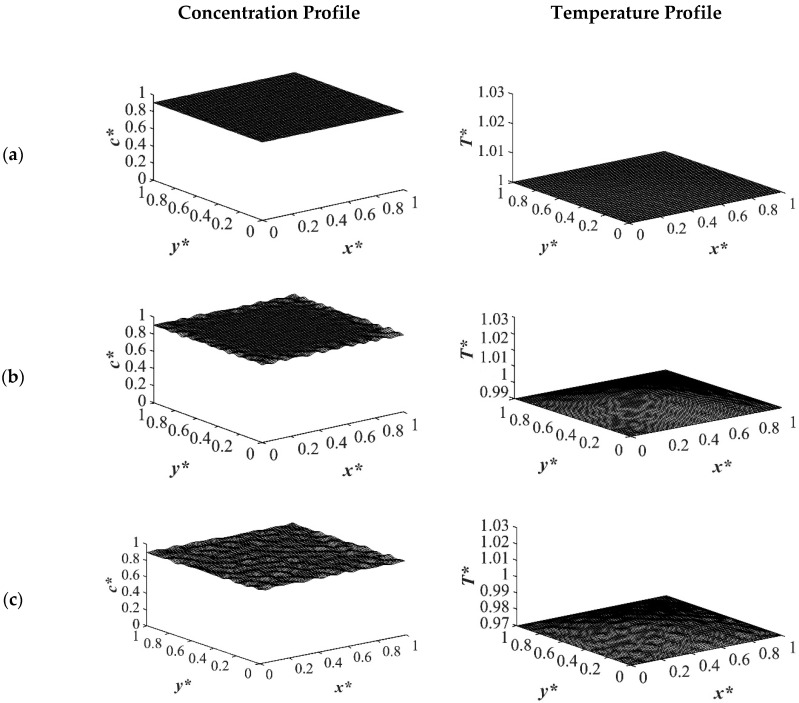
Spatial concentration (**left** column) and temperature (**right** column) profiles for the off-critical quench to c* = 0.909 at t* = 2.03 × 10^−5^ for three different quench depths of (**a**) T*_q1_ = 1.0, (**b**) T*_q2_ = 0.99 (**c**) T*_q3_ = 0.97.

**Figure 8:**
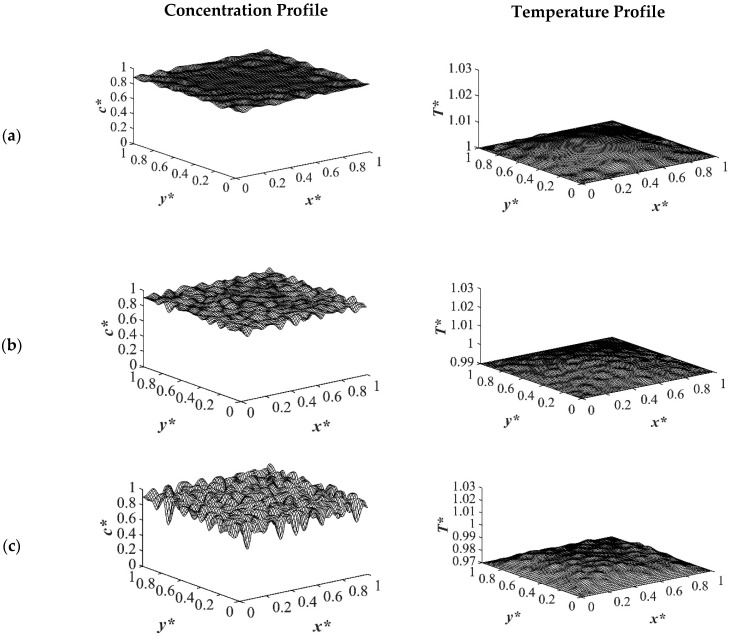
Spatial concentration (**left** column) and temperature (**right** column) profiles for the off-critical quench to c* = 0.909 at t* = 3.01 × 10^−4^ for three different quench depths of (**a**) T*_q1_ = 1.0, (**b**) T*_q2_ = 0.99 (**c**) T*_q3_ = 0.97.

**Figure 9:**
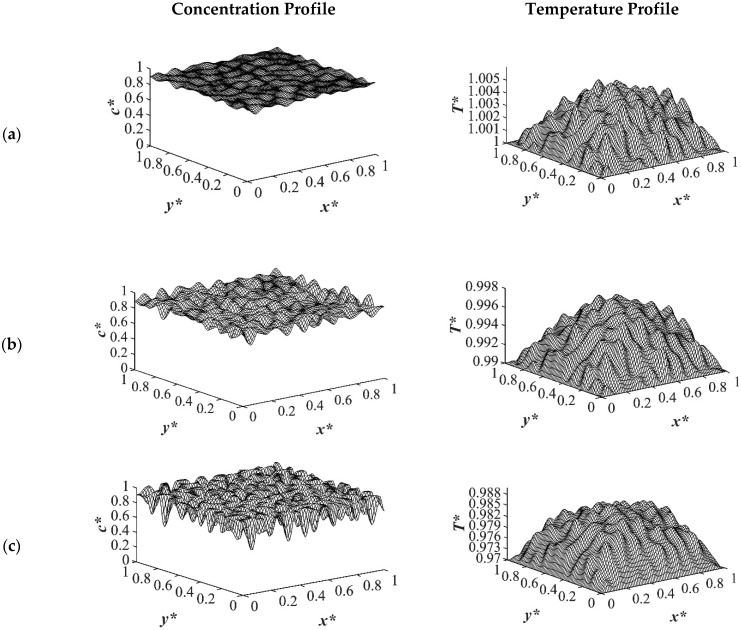
Spatial concentration (**left** column) and temperature (**right** column) profiles for the off-critical quench to c* = 0.909 at t* = 8.01 × 10^−4^ for three different quench depths of (**a**) T*_q1_ = 1.0, (**b**) T*_q2_ = 0.99 (**c**) T*_q3_ = 0.97.

The spatial dimensionless concentration profiles and the corresponding temperature profiles for case 2 in Table 1 of a critical quench with c* = 0.909 are presented in Figure 6, Figure 7, Figure 8 and Figure 9 for the same quench temperatures as case 1 to T*_q1_ = 1.0, T*_q2_ = 0.99, and T^*^_q3_ = 0.97 at different stages during phase separation. As presented in Figure 6 at t* = 1.03 × 10^−6^, the initial thermal concentration fluctuations existed in the two-phase region of the phase diagram where phase separation had not yet initiated, while the temperature profiles are different due to the difference in the heat transfer rates between the shallow and deep quenches. As the quench depth increased, the rate of heat transfer in the system also increased, which is reflected through the temperature profiles presented in Figure 6, Figure 7, Figure 8 and Figure 9. As shown in Figure 7, at t* = 2.03 × 10^−5^ for the shallow quench to T*_q1_ = 1.0, no phase separation was accomplished while heat transfer progressed considerably inside the sample and reached its homogenous state. On the other hand, as can be seen in Figure 7b,c, phase separation was initiated but was in the very early stage of phase separation with the temperature almost reaching its homogenous state and the entire sample. In the deep quench to T* _q3_ = 0.97, phase separation started and progressed inside the sample at t* = 2.03 × 10^−5^.

An interconnected morphology is formed due to the critical quench. The influence of the enthalpy of demixing started to emerge at Figure 7c where the system was quenched to T*_q3_ = 0.97. As time proceeded to t* = 3.01 × 10^−4^ as demonstrated in Figure 8, phase separation progressed in the entire sample and an interconnected morphology became apparent. As shown in the concentration profile in Figure 8c, phase separation progressed considerably compared to the shallow quench presented in Figure 8a,b. The corresponding temperature profile of each stage of phase separation reveals that even though the increase in the temperature due to the enthalpy of demixing is not substantial, its influence on the morphology formation during the thermally induced phase separation process cannot be neglected. At time equal to t* = 3.01 × 10^−4^, phase separation in the deep quench to T*_q3_ = 0.97 reached the late stage of phase separation and the medium started to coarsen from all four sides. The corresponding temperature profile also presented the highest increase in temperature. On the other hand, as presented in Figure 8a,b, phase separation was in the intermediate stage, with more phase separation being accomplished in the four boundaries where the quench was applied.

Figure 9 shows the phase separation and temperature profiles at t* = 8.01 × 10^−4^. The entire medium reached the late stage of spinodal decomposition and coarsened in the deep quench to T*_q3_ = 0.97 as presented in Figure 9a. Figure 9b presents the dimensionless concentration and temperature profiles of the quench temperature to T*_q2_ = 0.99. Compared to Figure 9a, more phase separation was carried out in the entire medium, and more heat was released due to phase separation. All the results provided for the case of critical quench, along with the results provided in Figure 2, Figure 3, Figure 4 and Figure 5, verify that deeper quench depths led to more phase separation with smaller droplet formation and a greater number of droplets. Besides, applying quench from four sides of the medium caused a gradient in the cooling rate between the sample boundaries and the interior parts, which led to anisotropic morphology formation.

**Figure 10:**
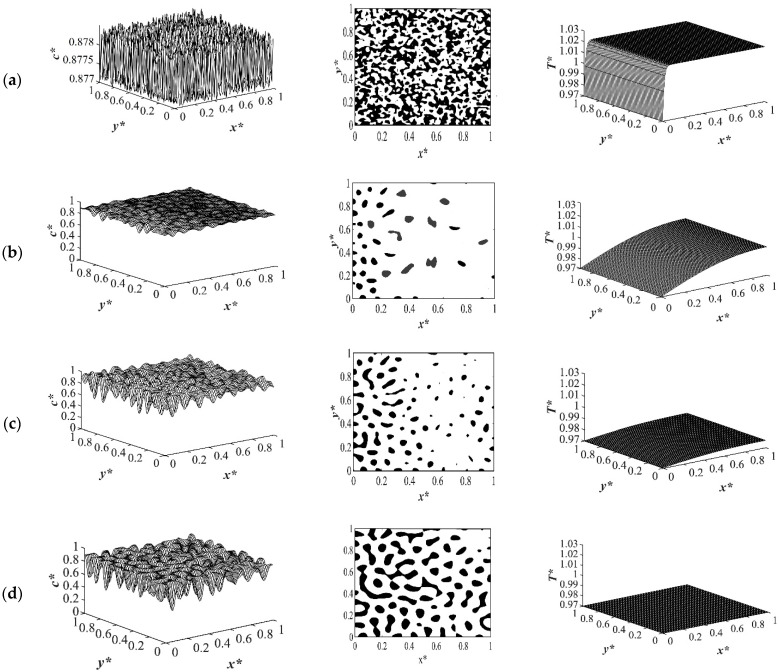
Spatial concentration (**left** column), phase separated pattern (**middle** column) and temperature (**right** column) profiles for off-critical quench without considering enthalpy of demixing c* = 0.88 at (**a**) t* = 1.03 × 10^−6^, (**b**) t* = 2.03 × 10^−5^, (**c**) t* = 8.03 × 10^−4^, and (**d**) t* = 1.03 × 10^−3^.

**Figure 11:**
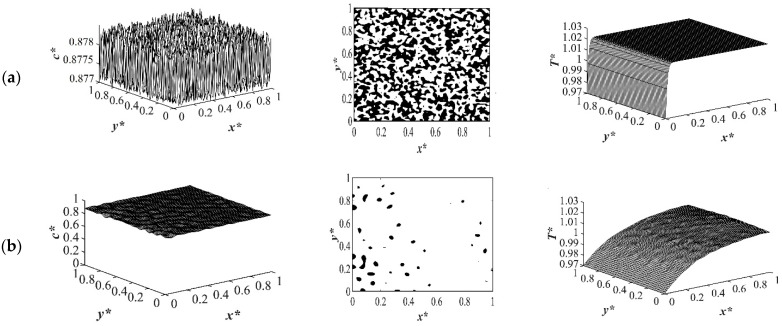

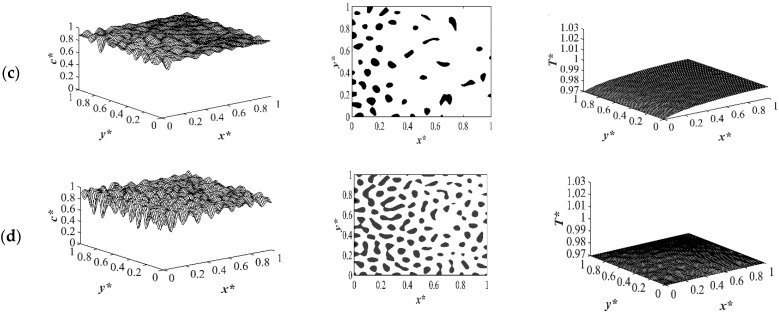
Spatial concentration (**left** column), phase separated pattern (**middle** column) and temperature (right column) profiles for off-critical quench considering enthalpy of demixing c* = 0.88 (**a**) t* = 1.03 × 10^−6^, (**b**) t* = 2.03 × 10^−5^, (**c**) t* = 8.03 × 10^−4^, and (**d**) t* = 1.03 × 10^−3^.

Case 3 in Table 1 provides the comparison between the phase separation with and without considering the enthalpy of demising when quenche was applied from one side of the medium while keeping the other sides insulated for an off-critical quench at c* = 0.88 to the dimensionless quench temperature of T*_q3_ = 0.97 as provided in Figure 10 and Figure 11, respectively. The first column represents the spatial concentration profile, the second column illustrates the phase-separated pattern, and the third column presents the corresponding spatial temperature profile. The white area presents the polymer-rich region where c* < 0.88 and the black area presents the solvent-rich region where c* > 0.88. The droplet-type morphology was formed due to the off-critical quench and the droplets on the left side of the medium where quench was applied merged and coarsened faster with time than the other sides of the medium that were insulated. The same initial composition, boundary conditions, and the quench rate were considered for both cases presented in Figure 10 and Figure 11.

Figure 10a and Figure 11a present the dimensionless concentration and temperature profiles after a very short time when quench was applied. The morphological pattern in these two figures reflect thermal fluctuations present in the solvent/polymer solution at early times. In both cases, phase separation did not start with thermal concentration fluctuations being present in the one-phase region of the phase diagram. Figure 10b and Figure 11b show the phase separated pattern and the corresponding temperature profile at time t* = 2.03 × 10^−5^. As it is shown, phase separation started in both cases from the left side of the sample where the quench was applied. However, the amount of the phase separation that was accomplished without considering the enthalpy of demixing was more than the case when the enthalpy of demixing was employed. This confirms the importance of considering the enthalpy of demixing during phase separation through spinodal decomposition because the heat increased the quench temperature to some extent and led to phase separation being accomplished at a higher temperature. Neglecting this parameter would cause loss of cost and energy during the industrial processes of porous polymeric materials’ formation.

The morphology that was formed was anisotropic with gradation in the pore size. The temperature profile presented in Figure 10b and 11b shows that the temperature had not reached its homogenous state yet and the phase separation in the quench side had started and was progressing inside the sample. The dimensionless concentration temperature profiles presented in Figure 10c and 11c show a later time during phase separation at t* = 8.03 × 10^−4^. Phase separation in the quench side progressed significantly, with smaller droplets forming in the quench side and larger droplets in the insulated sides. Considering Figure 10c and 11c confirms that the amount of the phase separation when the enthalpy of demixing was neglected was higher comparing to the case of considering it as presented in Figure 11c.

A closer look at both profiles shows that the number and the size of the droplets formed in the case where the enthalpy of demixing was considered was higher than in the case when it was neglected. This was due to the fact that the heat that was released during phase separation acted similar to applying a shallower quench to the system. Most of the heat is released during the early and intermediate stages of phase separation. This effect will decrease during the late stage of phase separation and diminish completely after the phase separation is completed. This was also confirmed previously in this study for the case of applying quench from four sides. As shown in Figure 10d and 11d, the droplets formed on the quench side entered the late stage of phase separation and started to coarsen. While on the insulated side, phase separation was still progressing. Allowing phase separation to proceed for a longer time would change the direction of anisotropy because the smaller droplets in the quench side would merge due to coarsening. At some specific time during phase separation, the morphology would resemble isotropic morphology, and after that time, the droplets on the quench side would become larger and the direction of anisotropy would change. In the late stage of phase separation, the droplets merged due to coarsening. Despite the fact that there are orders of magnitude difference between the experimental and theoretical phase separation growth rate, the results provided in this section are in good agreement with previous experimental and theoretical studies using polystyrene-cyclohexanol system [6,10,28,31,47,53,76,78].

**Figure 12:**
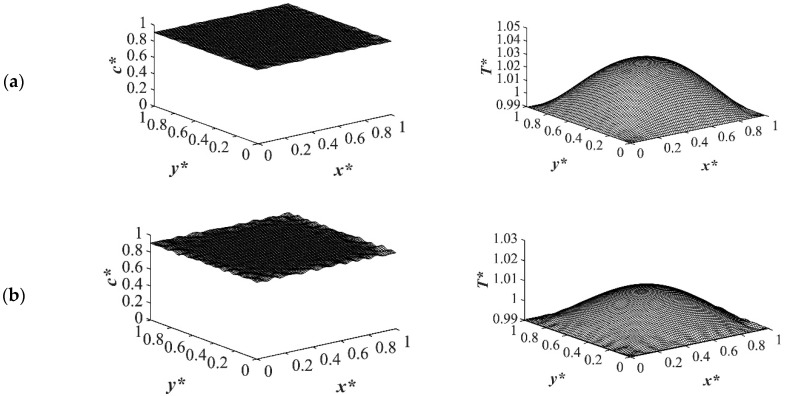
The influence of initial solution temperature on phase separation through TIPS process with two different dimensionless initial solution temperatures of (**a**) T* = 1.05 and (**b**) T* = 1.03 to the same quench temperature of T*_q2_ = 0.99 at time t* = 1.03 × 10^−5^.

**Figure 13:**
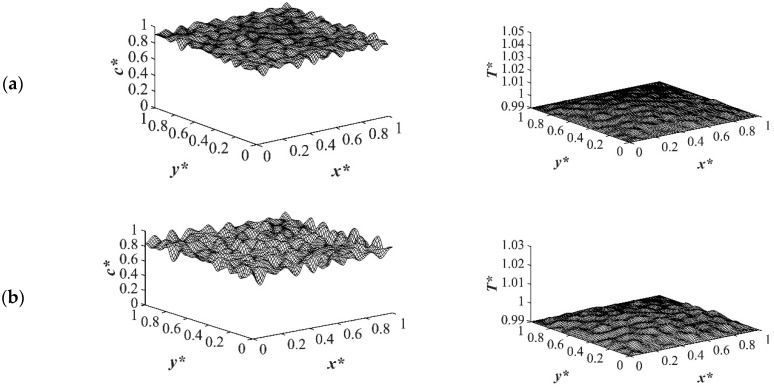
The influence of initial solution temperature on phase separation through TIPS process with two different dimensionless initial solution temperatures of (**a**) T* = 1.05 and (**b**) T* = 1.03 to the same quench temperature of T*_q2_ = 0.99 at time t* = 5.03 × 10^−4^.

The results of case 4 in Table 1 for the influence of initial solution temperature on the thermally induced phase separation process are provided in Figure 12 and Figure 13. The comparison between the phase separation starting from two different initial solution temperatures and its influence on morphology formation were evaluated. The dimensionless concentration and the corresponding temperature profile for an off-critical quench with the initial concentration of c* = 0.89 and the initial dimensionless temperature of T* = 1.05 and T* = 1.03 quenched to the same temperature of T*_q2_ = 0.99 are demonstrated. When the initial temperature of the solution was higher, as observed in Figure 12a it took more time for heat transfer to reach its homogenous state, which led to phase separation being accomplished at a higher temperature. Even if the quench was applied fast, the high viscosity of the solution prevented the temperature homogeneousness instantly. The droplet size was another factor that was influenced by the solution temperature. The high solution temperature led to longer quench time and, as a result, larger droplet formation as presented in the dimensionless concentration profiles in Figure 12. Comparing the dimensionless concentration profiles in Figure 12a,b, reveals that more phase separation was accomplished at the same time scale. Despite the fact that more heat was also released in the case presented in Figure 12b, the initial solution temperature was more predominant in increasing the phase separation temperature, which led to less phase separation. This effect was significant at the initial stages of phase separation when the phase separation was still in the early stage.

As time proceeded and phase separation progressed inside the sample at t* = 5.03 × 10^−4^, the amount of the heat generated in the initial solution temperature of T^*^ = 1.03 was larger than that in the case with the initial solution temperature of T* = 1.05 as presented in Figure 13b. After this time, when the amount of the enthalpy of demixing was competing with the initial solution temperature, the difference between the concentration profiles for the two cases of different solution temperatures could be neglected. Hence, it is of great importance to choose the most suitable initial solution temperature for phase separation based on the system properties and characteristics. If the enthalpy of demixing is high in a system, the initial solution temperature should be designated based on the competing effects between the heat generated in the system and the initial solution temperature as it directly influences the phase separating morphology.

## 4. Conclusions

In this study, the influence of spatial and temporal temperature gradients on the thermally induced phase separation process was investigated through modelling and simulation in two- dimensions by coupling the nonlinear Cahn-Hilliard equation considering all temperature-dependent parameters and the Fourier heat transfer equation considering the influence of enthalpy of demixing. The Flory-Huggins free energy theory, the Rouse model, and slow mode theory were employed for the unentangled polymer chain diffusion. Different boundary conditions, initial polymer concentrations, and quench depths were evaluated. It was verified that the influence of heat transfer on phase separation could not be neglected. This was due to the fact that the morphology that was formed differed due to the interaction and the influence of heat transfer on phase separation. Results obtained in this study showed that the quench depth had a significant impact on the phase separated morphology in terms of the time required for phase separation and, to a lesser extent, the morphology. Applying quench from one side or from all four sides of the sample led to anisotropic morphology formation as a result of variation in the quench rate. Comparing the cases applying the enthalpy of demixing and without the heat generation term revealed that the heat that was released during phase separation led to phase separation to proceed at a higher temperature than the initial quench temperature. Even though the amount of the enthalpy of demixing is not high for polymer solutions, its influence on the morphology formation could not be neglected.

## Figures and Tables

**Table 1:** Conditions studied in this paper including boundary condition, quench depths, and initial average concentrations of the solvent.

Case	$c^{*}$	$T^{*}$	$T^{*}{}_{q1}$	$T^{*}{}_{q2}$	$T^{*}{}_{q3}$	Boundary Conditions
**1**	0.85	1.03	1.0	0.99	0.97	$T^{*}=T^{*}{}_{q}$ $\;\mathrm{at}\;t^{*}>0$ $\;\mathrm{and}\;x^{*}=0$ $T^{*}=T^{*}{}_{q}$ $\;\mathrm{at}\;t^{*}>0$ $\;\mathrm{and}\;x^{*}=1$ $T^{*}=T^{*}{}_{q}$ $\;\mathrm{at}\;t^{*}>0$ $\;\mathrm{and}\;y^{*}=0$ $T^{*}=T^{*}{}_{q}$ $\;\mathrm{at}\;t^{*}>0$ $\;\mathrm{and}\;y^{*}=1$
**2**	0.909	1.03	1.0	0.99	0.97	$T^{*}=T^{*}{}_{q}$ $\;\mathrm{at}\;t^{*}>0$ $\;\mathrm{and}\;x^{*}=0$ $T^{*}=T^{*}{}_{q}$ $\;\mathrm{at}\;t^{*}>0$ $\;\mathrm{and}\;x^{*}=1$ $T^{*}=T^{*}{}_{q}$ $\;\mathrm{at}\;t^{*}>0$ $\;\mathrm{and}\;y^{*}=0$ $T^{*}=T^{*}{}_{q}$ $\;\mathrm{at}\;t^{*}>0$ $\;\mathrm{and}\;y^{*}=1$
**3**	0.88	1.03	$T^{*}{}_{q}=0.97$			$T^{*}=T^{*}{}_{q}$ $\;\mathrm{at}\;t^{*}>0$ $\;\mathrm{and}\;x^{*}=0$ $\frac{\partial T^{*}}{\partial x}=T^{*}{}_{q}$ $\;\mathrm{at}\;t^{*}>0$ $\;\mathrm{and}\;x^{*}=1$ $\frac{\partial T^{*}}{\partial y}=T^{*}{}_{q}$ $\;\mathrm{at}\;t^{*}>0$ $\;\mathrm{and}\;y^{*}=0$ $\frac{\partial T^{*}}{\partial y}=T^{*}{}_{q}$ $\;\mathrm{at}\;t^{*}>0$ $\;\mathrm{and}\;y^{*}=1$
			(a) With enthalpy of demixing		(b) Without enthalpy of demixing	
**4**	0.89	$T^{*}{}_{1}$	$T^{*}{}_{2}$		$T^{*}{}_{q}$	$T^{*}=T^{*}{}_{q}$ $\;\mathrm{at}\;t^{*}>0$ $\;\mathrm{and}\;x^{*}=0$ $T^{*}=T^{*}{}_{q}$ $\;\mathrm{at}\;t^{*}>0$ $\;\mathrm{and}\;x^{*}=1$ $T^{*}=T^{*}{}_{q}$ $\;\mathrm{at}\;t^{*}>0$ $\;\mathrm{and}\;y^{*}=0$ $T^{*}=T^{*}{}_{q}$ $\;\mathrm{at}\;t^{*}>0$ $\;\mathrm{and}\;y^{*}=1$
		1.03	1.05		0.99	

## Data Availability

The data presented in this study are available on request from the corresponding author.
